# Characterization of the Endothelial Cell Cytoskeleton following HLA Class I Ligation

**DOI:** 10.1371/journal.pone.0029472

**Published:** 2012-01-11

**Authors:** Mary E. Ziegler, Puneet Souda, Yi-Ping Jin, Julian P. Whitelegge, Elaine F. Reed

**Affiliations:** 1 The Department of Pathology and Laboratory Medicine, David Geffen School of Medicine, University of California Los Angeles, Los Angeles, California, United States of America; 2 The Pasarow Mass Spectrometry Laboratory, The Semel Institute for Neuroscience and Human Behavior, David Geffen School of Medicine, University of California Los Angeles, Los Angeles, California, United States of America; University of Birmingham, United Kingdom

## Abstract

**Background:**

Vascular endothelial cells (ECs) are a target of antibody-mediated allograft rejection. *In vitro*, when the HLA class I molecules on the surface of ECs are ligated by anti-HLA class I antibodies, cell proliferation and survival pathways are activated and this is thought to contribute to the development of antibody-mediated rejection. Crosslinking of HLA class I molecules by anti-HLA antibodies also triggers reorganization of the cytoskeleton, which induces the formation of F-actin stress fibers. HLA class I induced stress fiber formation is not well understood.

**Methodology and Principal Findings:**

The present study examines the protein composition of the cytoskeleton fraction of ECs treated with HLA class I antibodies and compares it to other agonists known to induce alterations of the cytoskeleton in endothelial cells. Analysis by tandem mass spectrometry revealed unique cytoskeleton proteomes for each treatment group. Using annotation tools a candidate list was created that revealed 12 proteins, which were unique to the HLA class I stimulated group. Eleven of the candidate proteins were phosphoproteins and exploration of their predicted kinases provided clues as to how these proteins may contribute to the understanding of HLA class I induced antibody-mediated rejection. Three of the candidates, eukaryotic initiation factor 4A1 (eIF4A1), Tropomyosin alpha 4-chain (TPM4) and DDX3X, were further characterized by Western blot and found to be associated with the cytoskeleton. Confocal microscopy analysis showed that class I ligation stimulated increased eIF4A1 co-localization with F-actin and paxillin.

**Conclusions/Significance:**

Colocalization of eIF4A1 with F-actin and paxillin following HLA class I ligation suggests that this candidate protein could be a target for understanding the mechanism(s) of class I mediated antibody-mediated rejection. This proteomic approach for analyzing the cytoskeleton of ECs can be applied to other agonists and various cells types as a method for uncovering novel regulators of cytoskeleton changes.

## Introduction

The composition of the eukaryotic cytoskeleton consists mainly of three discrete fibrous structures—actin microfilaments, microtubules and intermediate filaments. These structures provide the framework to maintain cell shape and polarity and also contribute to other fundamental cellular functions such as motility, organelle transport and division [1]. These cellular processes are carried out through the actions of multiple molecules, which comprise functional modules [2]. These interactions can been seen in cryoelectron tomography images, in which functional modules appear to be spatially congested while at the same time highly organized [3], [4]. It has been suggested that an intact actin cytoskeleton is fundamentally responsible for the functional organization within the cell [5].

In ECs, the cytoskeleton plays an essential role in providing a sturdy intracellular scaffold, which serves to organize vital membrane proteins within the cell. The cytoskeleton also has the ability to respond to extracellular stimuli and undergo reorganization [6]. Additionally, several lines of evidence support the notion that numerous soluble proteins can be associated with the cytoskeleton [5]. These findings boost the role of the cytoskeleton, as not only the defining structure of the cell, but also as a key regulator of the intracellular environment [7].

When ECs are quiescent, actin creates a cortical ring providing a link between cell-cell and cell-matrix adhesion complexes and intracellular organelles. The cortical actin ring is necessary to sustain the EC barrier function [8] and its disruption can initiate EC permeability [9]. When ECs are stimulated with inflammatory agonists such as thrombin and histamine, the cortical actin ring is reorganized in such a manner that stress fibers are created, which extend throughout the cell [10].

Understanding how ECs respond to stimuli that mediate cytoskeleton changes is an important topic in organ allograft rejection. Antibodies directed toward HLA class I molecules on the donor endothelium play a major role in antibody-mediated transplant rejection [11]. Several studies suggest that the signaling events that occur in EC following the binding of HLA class I antibodies contribute to the process of chronic rejection and transplant vasculopathy. Signaling through the HLA class I molecules on the surface of ECs is associated with changes in the actin cytoskeleton. HLA class I ligation on ECs activates the GTP binding protein RhoA [12] and utilizes Rho GTPase and Rho-kinase (ROK) to promote stress fiber formation [13]. In addition, HLA class I ligation activates Src, focal adhesion kinase (FAK), and paxillin [14]. FAK is central regulator of this signaling cascade as it is critical for focal adhesion assembly and stress fiber formation [15]. These signaling cascades represent early events leading to activation of downstream pathways in the endothelium that can be detrimental to graft survival. One such pathway is the proliferation pathway. The mammalian target of rapamycin (mTOR) is an essential regulator of cell proliferation and protein synthesis through the activation of p70 ribosomal S6 kinase (S6K) and eukaryotic initiation factor 4E-binding protein 1 (4E-BP1) [16], [17]. Ligation of HLA class I molecules on the surface of ECs promotes proliferation via the mTOR pathway [18]. Understanding how cytoskeleton changes contribute to these signaling cascades will advance our understanding of EC proliferation, migration and permeability, all of which may contribute to the mechanism(s) underlying antibody-mediated transplant rejection.

Here we present the results of a proteomic-based analysis characterizing changes in the cytoskeleton of human aortic ECs following stimulation with thrombin, basic fibroblast growth factor and HLA class I antibodies. The contents of these isolations were characterized by mass spectrometry, annotated and a candidate list of HLA class I-induced cytoskeleton associated proteins was identified. Three of the candidates, TPM4, eIF4A1 and DDX3X, were further characterized. TPM4 was uniquely found in the cytoskeletal fraction of class I stimulated ECs, but absent in the cytoskeleton of ECs stimulated with thrombin and bFGF. The eIF4A1 protein, a regulator of protein synthesis and cell proliferation, was found in association with the actin cytoskeleton by Western blot and showed a higher degree of colocalization with F-actin and paxillin in HLA class I stimulated cells compared to the other treatment groups. This proteomic discovery approach provides candidate targets, which may link HLA class I-induced cytoskeleton changes to downstream cellular functions such as proliferation.

## Materials and Methods

### Ethics Statement

Informed written consent for use of the aortic tissue as an anatomical gift for research was obtained by OneLegacy (a federally-designated organ procurement organization) at the time of organ donation from the next of kin or authorized party. The use of the human aortic tissue for the research described herein was approved by the OneLegacy Biomedical Review Board under the agreement #RS-02-10-2 and UCLA MTA2009-561.

### Antibodies and Chemicals

Cytochalasin D was purchased from Sigma-Aldrich. Recombinant human bFGF was purchased from R&D Systems. Human α-thrombin was from Enzyme Research Laboratories. The murine monoclonal antibody W6/32 (IgG2a), reactive with a monomorphic epitope on HLA class I antigens, was obtained from the American Type Culture Collection. The mouse IgG used as an isotype control was supplied by Sigma-Aldrich. Recombinant human FGF basic was from R&D Systems. Antibodies used for Western blot were β-actin, eIF4A1, 4E-BP1 obtained from Cell Signaling and vinculin, β-tubulin, and myosin light chain (all three from Santa Cruz) and β1-integrin and paxillin (BD Biosciences) and TPM4 and DDX3X (both from Acris Antibodies). The eIF4A1 antibody used for confocal microscopy was obtained from Santa Cruz. A second eIF4A1 antibody obtained from Abcam was used to confirm the specificity of the immunofluorescent staining pattern. The fluoresent conjugated secondary antibodies, Alexafluor 488 goat anti-rabbit, Rhodamine Red™-X goat anti-mouse and Texas Red-phalloidin were from Invitrogen.

### Cell Culture

Primary human aortic ECs were isolated from the aortic rings of explanted donor hearts, as previously described [19]. ECs were cultured in M199 complete medium composed of sodium pyruvate (1 mM) (Irvine Scientific), penicillin (100 U/mL) and streptomycin (100 µg/ml) (both from Invitrogen), 20% (v/v) FBS (HyClone), heparin (90 µg/ml) (Sigma-Aldrich), and endothelial cell growth supplement (20 µg/ml) (Fisher Scientific). Cells from passages 4–10 were used at 80%–90% confluence.

### F-actin Staining

ECs grown to 80–90% were starved for 2 h in basal medium containing 0.2% FBS followed by treatment with mAb W6/32 (1 µg/ml) (referred to as HLA class I stimulated), isotype control mouse IgG (1 µg/ml) (referred to as unstimulated), thrombin (1 U/ml) or bFGF (25 ng/ml) for 10 min. Treated ECs were fixed with 4% paraformaldehyde and permeabilized with 0.2% Triton-X-100. The presence of F-actin was visualized by direct staining with Texas Red–phalloidin. Cells were analyzed with Zeiss Axioplan 2 microscope with the Zeiss FluoArc 100 watt mercury light source. Digital images of fluorescence were obtained with a cooled charge-coupled device camera (SPOT 2, Diagnostic Instruments) and associated software (SPOT 4.5, Diagnostic Instruments).

### Cytoskeleton Isolation

The method was adapted from a previously published method [7]. Prior to use in experiments, ECs were grown for 16 h in medium containing 0.2% FBS. ECs were treated with mAb W6/32 (1 µg/ml), mouse isotype control IgG (1 µg/ml), thrombin (1 U/ml) or bFGF (25 ng/ml) for 10 min at 37°C, washed once with ice-cold PBS and lysed in buffer containing (20 mM Tris (pH 7.9), 137 mM NaCl, 5 mM EDTA, 1 mM EGTA, 10% glycerol, 1% Triton X-100, 10 mM NaF, 1 mM PMSF, 1 mM Na3VO4, 10 µg/ml aprotinin, and 10 µg/ml leupeptin) for 10 min on ice. The samples were sonicated for 30 sec. Tosylactivated Dynal-beads M280 (50 µl/sample) (Invitrogen) were first pre-blocked using a blocking buffer (0.5 M Tris, 1 M glycine). Next the beads were washed once with PBS. The lysate was then added to the beads and the mixture incubated on ice for 15 min with intermittent mixing. The beads were collected by a magnetic particle concentrator (MPC) (Dynal) and washed 2 times with PBS and 2 times with 10 mM Tris-HCl pH 8.

### Western Blot

Beads containing the cytoskeleton isolation preparations were heated for 5 min at 95°C in SDS sample buffer, electrophoresed on SDS polyacrylamide gels, and transferred to a polyvinylidene difluoride membrane. The membranes were blocked using 5% nonfat dry milk in TBS (pH 7.4) containing 0.1% Tween 20 (TBST) for 20 min at room temperature, and incubated with the appropriate primary antibody overnight at 4°C. The blots were washed with TBST followed by incubation in HRP-conjugated secondary antibody (Santa Cruz Biotechnology) for 1 h at room temperature. The blots were subsequently washed with TBST and developed with ECL.

### In-Solution Tryptic Digestion

Beads containing the cytoskeleton proteins were resuspended in digestion buffer (50 mM ammonium bicarbonate). The sample was first reduced using 100 mM DTT (Sigma-Aldrich). Next, the preparation was alkylated using 100 mM iodoacetamide (Sigma Aldrich). The proteins were then digested overnight using proteomics grade trypsin (Sigma Aldrich) at a concentration of 0.1 µg/µl. Following trypsin digest, the beads were centrifuged at 14,000× g for 1 min and the supernatant was collected.

### Peptide Purification

Magnetic Dynabeads RPC 18 (Invitrogen) were used to bind the cytoskeleton peptides for purification. RPC 18 beads were first washed with binding buffer (0.1% TFA/0.5%ACN). The cytoskeleton peptides were mixed with binding buffer then added to the beads. The beads were washed 4 times with binding buffer and eluted with 60% ACN.

### Nano-liquid chromatography with tandem mass spectrometry (nLC-MSMS)

nLC-MS/MS with Collision Induced Dissociation (CID) is performed on a 7 tesla LTQ FT Ultra (Thermo Scientific, Waltham, MA) integrated with an Eksigent nano-LC. A prepacked reverse-phase column (Microtech Scientific C18 with a dimension of 100 µm×3.5 cm) containing reverse-phase resin (Biobasic C18, 5-µm particle size, 300-Å pore size, Microtech Scientific, Fontana, CA) is used for peptide chromatography and subsequent CID analyses. ESI conditions using the nano-spray source (Thermo Fisher) for the LTQ-FT are set as follows: capillary temperature of 220°C, tube lens 110 V and a spray voltage of 2.5 kV. The flow rate for reverse-phase chromatography is 5 µl/min for loading and 300 nl/min for the analytical separation (buffer A: 0.1% formic acid, 1% ACN; buffer B: 0.1% formic acid, 100% ACN). Peptides are resolved by the following gradient: 2–60% buffer B over 40 min, then increased to 80% buffer B over 10 min and then returned to 0% buffer B for equilibration of 10 min. The LTQ FT is operated in data-dependent mode with a full precursor scan at high-resolution (100000 at m/z 400) and six MSMS experiments at low resolution on the linear trap while the full scan is completed. For CID the intensity threshold was set to 5000, where mass range was 350–2000. Spectra are searched using Mascot software (Matrix Science, UK) in which results with p<0.05 (95% confidence interval) were considered significant and indicating identity. The data was also analyzed through Sequest database search algorithm implemented in Discoverer software (Thermo Fisher, Waltham, MA).

### Confocal Microscopy

ECs were grown on 35 mm glass bottom dishes to 80–90% confluence and starved for 2 h in basal medium containing 0.2% FBS followed by treatment with mAb W6/32 (1 µg/ml), isotype control mouse IgG (1 µg/ml), thrombin (1 U/ml) or bFGF (25 ng/ml) for 10 min. Treated ECs were fixed with 4% paraformaldehyde and permeabilized with 0.2% Triton-X-100. Cells were incubated with primary antibody (mouse anti-paxillin or rabbit anti- eIF4A1) in 5% BSA in PBS overnight at 4°C washed and incubated with secondary antibody (Alexafluor 488 goat anti-rabbit for eIF4A1 and Rhodamine Red™-X goat anti-mouse IgG for paxillin) for 30 min at room temperature. The presence of F-actin was visualized by direct staining with Texas Red–phalloidin. Cell images were captured using a Zeiss LSM 510 confocal microscope at 63× magnification using the Zeiss LSM 5 PASCAL software (Carl Zeiss MicroImaging GmbH, Germany)

## Results

### HLA class I ligation induces stress fiber formation

**Figure 1:**
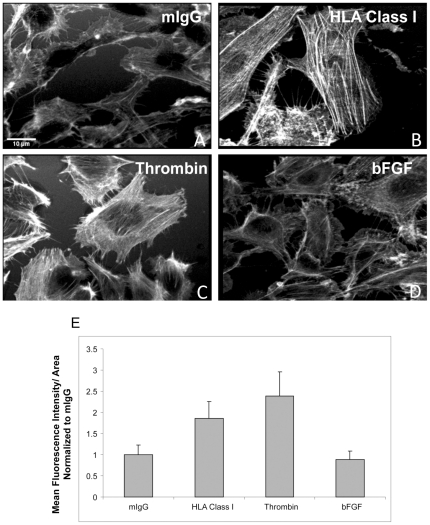
Comparison of HLA class I stimulated EC cytoskeleton changes with other agonists. Human aortic ECs were treated with either mIgG 1 µg/ml (A), HLA class I antibody (Ab) 1 µg/ml (B), thrombin 1 U/ml (C) or bFGF 25 ng/ml (D) for 10 min. Cells were stained with Texas-Red–phalloidin and analyzed by fluorescence microscopy. (E) The fluorescence intensity and the total area of each cell in each field were measured using ImageJ software (http://rsb.info.nih.gov/ij/). Three independent experiments were performed and in each experiment 3 fields were measured. The ratio between the mean fluorescence intensity and the cell area was calculated to account for changes in cell shape due to contraction. The data was normalized to the mIgG group and the others groups were calculated to reflect the change compared to the mIgG group. A student's t-test was performed to determine significant changes between the groups, P<0.05.

A comparison of F-actin reorganization by immunofluorescence revealed that stimulation with HLA class I antibodies (Fig. 1B) or thrombin (Fig. 1C) activated stress fiber formation, while bFGF (Fig. 1D) failed to produce stress fibers. Stimulation of ECs with an antibody to HLA class I showed an 86% increase in the mean fluorescence intensity of F-actin staining (p = 5E-07). Thrombin stimulation also significantly increased the mean fluorescence intensity of F-actin staining by 139% (p = 2E-09), whereas stimulation with bFGF decreased F-actin staining by 11% compared to the unstimulated cells (Fig. 1E). Although both thrombin and HLA class I antibodies triggered actin remodeling, the HLA class I antibody-induced stress fiber response was weaker than that induced by thrombin (Fig. 1C vs 1B). We examined the effects of low and high doses of thrombin and compared it to the signaling pathways elicited by anti-HLA class I antibodies (MZ unpublished data). Stimulation with thrombin at high concentrations (1 U/ml) induced a robust increase in the intracellular Ca^2+^ concentration and promoted stress fiber formation in an ERK-independent manner. In contrast, stimulation of ECs with either HLA class I antibodies or a low dose of thrombin (1 mU/ml) did not promote any detectable change in intracellular Ca^2+^ concentration, but induced myosin light chain (MLC) phosphorylation and stress fiber assembly via MLC kinase (MLCK) and ROK in an ERK1/2-dependent manner. Thus, the pathway activated by the high dose of thrombin is different from the pathway activated by a lower dose of thrombin and HLA class I ligation. Given these observations, we hypothesized that the isolation and proteomic characterization of the cytoskeletal proteins in HLA class I stimulated ECs could lead to a better understanding of the mechanisms underlying HLA class I induced stress fibers.

### The EC cytoskeleton can be isolated

**Figure 2:**
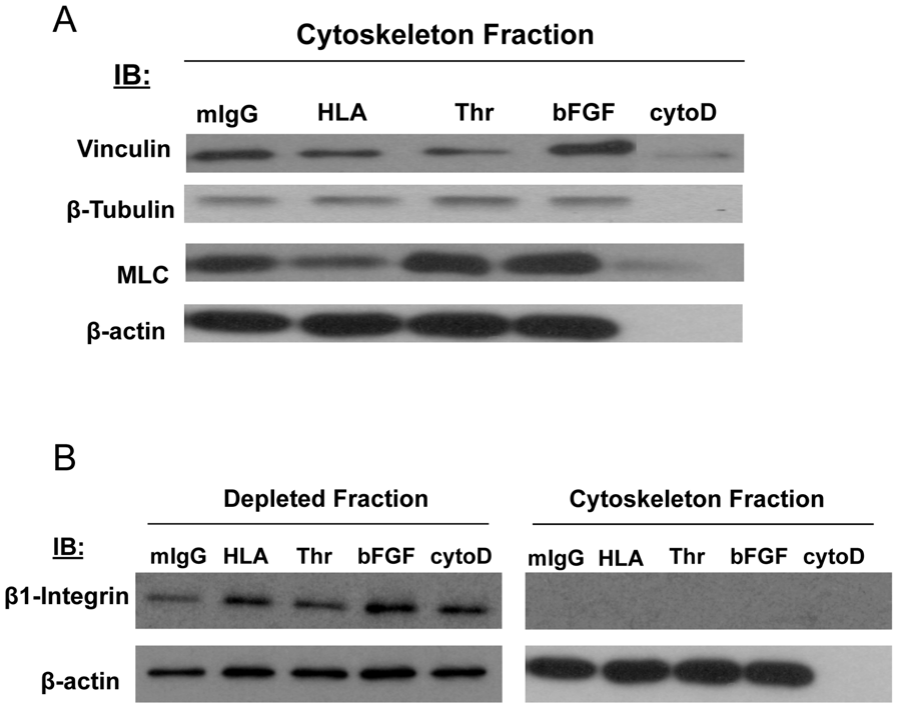
Validation of the EC cytoskeleton isolation by Western blot. (A) Human aortic ECs were treated for 10 min with either mIgG 1 µg/ml (lane 1), HLA class I Ab 1 µg/ml (lane 2), thrombin 1 U/ml (lane 3), bFGF 25 ng/ml (lane 4) or treated with cytochalasin D 5 µM (lane 5) for 30 min. The cytoskeleton was isolated from the lysates using tosylactived magnet Dynal beads and the fraction was analyzed by Western blot for components of the cytoskeleton. (B) Human aortic ECs were treated for 10 min with either mIgG 1 µg/ml (lanes 1 and 6), HLA class I Ab 1 µg/ml (lanes 2 and 7), thrombin 1 U/ml (lanes 3 and 8), bFGF 25 ng/ml (lanes 4 and 9) or treated with cytochalasin D 5 µM (lanes 5 and 10) for 30 min. The cytoskeleton was isolated using tosylactived magnetic Dynal beads. The cytoskeleton fraction (lanes 6–10) and the depleted fraction after cytoskeleton isolation (lanes 1–5) were immunoblotted to detect the presence of ß1-integrin and ß-actin as a loading control. The data presented in panels A and B are representative of 5 independent experiments.

Previous studies showed that the cytoskeleton and its associated proteins could be selectively enriched using Dynal beads [7]. The cytoskeleton was isolated from unstimulated ECs and ECs stimulated with an HLA class I antibody, thrombin or bFGF. For the following Western blot experiments, ECs were also treated with a cytochalasin D followed by cytoskeleton isolation. Western blot analysis revealed that when an intact cytoskeleton was isolated from ECs, the cytoskeleton proteins vinculin, ß-tubulin, MLC and ß-actin were detected, while disruption of the cytoskeleton with cytochalasin D prevented the isolation of these proteins (Figure 2A). Previous studies demonstrated that the absence of ß1 integrin in the cytoskeleton fraction marks the selective enrichment of the cytoskeleton and its associated proteins [7], [20]. When the cytoskeleton was isolated from ECs, the ß1 chain of integrin was only detected in the cytoskeleton-depleted fraction and was not found in the cytoskeleton-enriched fraction (Figure 2B).

### Identification of cytoskeleton associated proteins

Using in-solution trypsin digestion the isolated cytoskeleton fraction from each treatment group was digested into peptides. To identify the proteins in the cytoskeleton isolation preparations, nLC-MS/MS was performed on the peptides and Mascot searches were carried out. The list of proteins identified in each treatment group is summarized in Table S1. A total of 128 cytoskeleton-associated proteins were identified in unstimulated ECs, 126 in HLA class I stimulated ECs, 67 in thrombin treated ECs and 88 in bFGF treated ECs. When these proteins were compared between the treatment groups we identified 62 unique proteins in the unstimulated group, 56 in the HLA class I group, 10 in the thrombin group and 20 in the bFGF group.

### Classification of the cytoskeleton proteins

**Figure 3:**
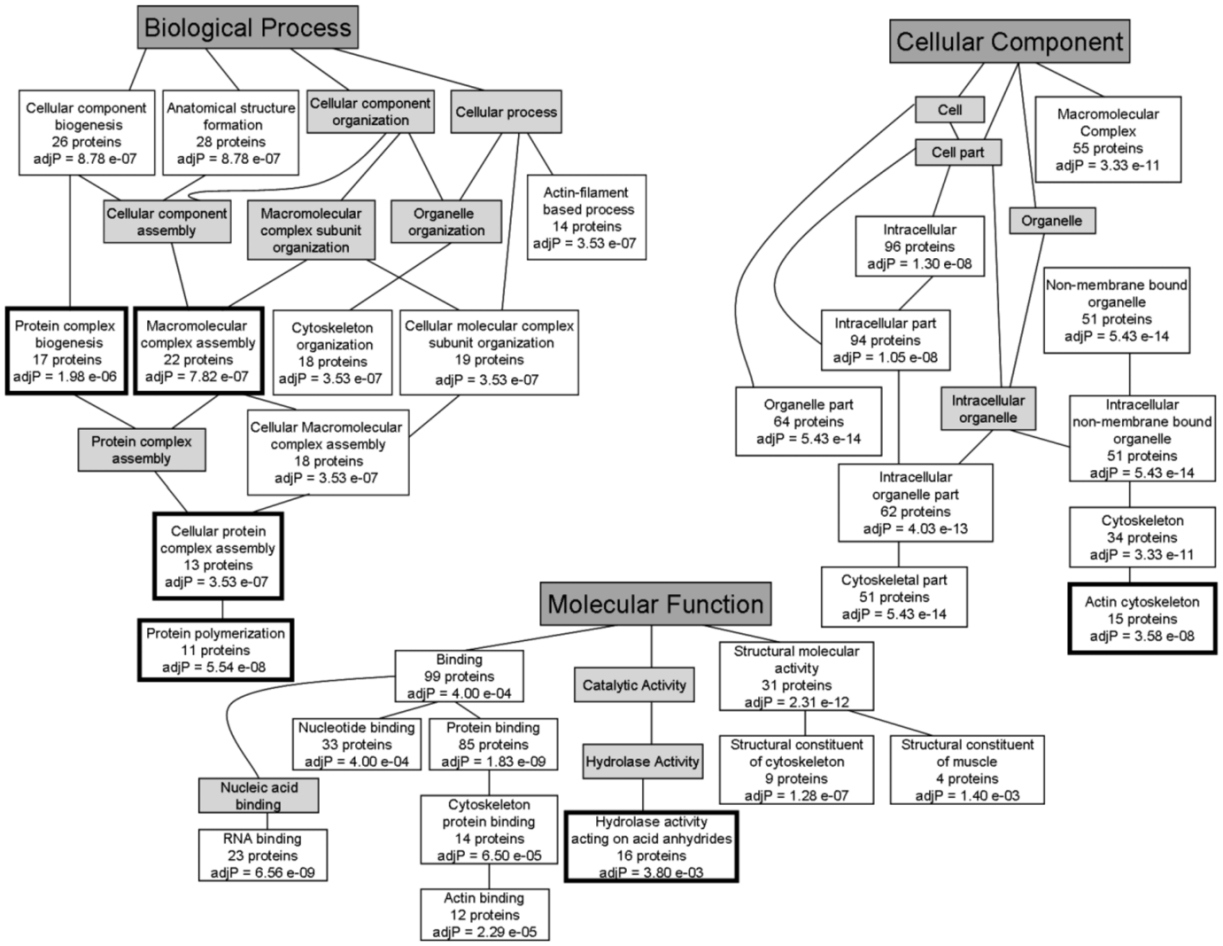
Directed acyclic graph (DAG) representing the enriched GO categories in the HLA class I stimulated EC cytoskeleton isolation. The Gene Ontology (GO) enrichment analysis tool called Gene Onology Tree Machine (GOTM) was used to create the DAG. The gene list was created using the uniprot accession numbers and thus the gene ID type selected was “hsapiens_uniprot_swissprot_accession.” Next the reference for GOTM was selected as “hsapiens_genome.” The statistical method was set to the default “hypergeometric,” the multiple test adjustment was set to be done by Benjamini & Hochberg, the significance level was set to the top 10 and the minimum number of genes for a category was 2. The DAG is a re-creation of the graph generated by GOTM. The shaded boxes indicate categories that were not significantly enriched. The significantly enriched categories found in the HLA class I treated group are specified by the white boxes and include the adjusted p-values and the number of proteins found in each category. The categories found to be uniquely enriched only by HLA class I stimulation are indicated in bold.

The identified proteins were classified using the Gene Ontology (GO) enrichment analysis tool Gene Onology Tree Machine (GOTM) [21]. The results are represented as a directed acyclic graph (DAG) in order to preserve the relationships among the enriched GO categories. For each treatment group, a DAG was produced and certain GO categories were significantly enriched in each data set. Figure 3 is a re-creation of the DAG generated by GOTM for the HLA class I stimulated group. The grey boxes are categories that were not significantly enriched, while the significantly enriched categories found in the HLA class I treated group are shown by the white boxes and include the adjusted p-values (using Benjamini Hochberg) and the number of proteins found in each category. The significantly enriched categories in the HLA class I treated group were compared to the other treatment groups and if the categories in the class I group were also enriched in any of the other treatment groups then these categories were not considered a unique category of HLA class I unique cytoskeleton changes. The categories found to be uniquely enriched by HLA class I stimulation are indicated by the bold-faced boxes (Fig. 3).

To determine which proteins are important for inducing HLA class I cytoskeleton changes, the proteins found under the unique categories of the HLA class I DAG were examined (Fig. 3, bold-faced boxes). Using this list of proteins in the HLA class I group, each protein was searched in the original list of unstimulated, thrombin and bFGF treated cells (Table S1). If a protein found under the HLA class I unique category was also found in any of the other treatment groups it was not considered a candidate protein. After eliminating the proteins that were also found in the other treatments groups, 12 candidate proteins were identified (Table 1).

**Table 1:** 12 Candidate Proteins Representing HLA Class I Induced Cytoskeleton Changes.

Accession	Protein Name	Gene Name	GO Categories
Q8WZ74	Cortactin-binding protein 2	CTTNBP2	protein polymerization
			cellular protein complex assembly
			macromolecular complex assembly
			protein complex biogenesis
O15145	Actin-related protein 2/3 complex subunit 3	ARPC3	protein polymerization
			cellular protein complex assembly
			macromolecular complex assembly
			protein complex biogenesis
			actin cytoskeleton
Q99880	Histone H2B type 1-L	HIST1H2BL	macromolecular complex assembly
P83731	60S ribosomal protein L24	RPL24	macromolecular complex assembly
P04114	Apolipoprotein B-100	APOB	macromolecular complex assembly
P11142	Heat shock cognate 71 kDa protein	HSPA8	hydrolase activity, acting on acid anhydrides
O00571	ATP-dependent RNA helicase DDX3X	DDX3X	hydrolase activity, acting on acid anhydrides
P60842	Eukaryotic initiation factor 4A-I	EIF4A1	hydrolase activity, acting on acid anhydrides
P35249	Replication factor C subunit 4	RFC4	hydrolase activity, acting on acid anhydrides
P49790	Nuclear pore complex protein Nup153	NUP153	hydrolase activity, acting on acid anhydrides
P06576	ATP synthase subunit beta, mitochondrial	ATP5B	hydrolase activity, acting on acid anhydrides
P67936	Tropomyosin alpha-4 chain	TPM4	actin cytoskeleton

### Prediction of protein kinases

Among the 12 candidates identified, 11 of the proteins were phosphoproteins with the exception of RCF4 (Table 1). The 11 candidate phosphoprotein sequences and their corresponding phosphorylation sites were submitted to the online-software NetworKIN-2.0, which provides a list of kinase predictions and grants insight into the interaction networks between phosphoproteins and kinase families based on the latest human phosphoproteome [22]. Using the known phosphorylation sites of the 11 candidate phosphoproteins it was determined that 35 kinase families could be responsible for their activation and among these 35 families there were 70 individual kinases (Table 2).

**Table 2:** Predicted Kinase Families and the Names of Distinct Kinases and Candidate Target Phosphoproteins in Each Family.

Kinase Family	Kinases	Candidate Phosphoproteins
ACTR2_ACTR2B_TGFbR2	TGF-beta type II receptor	HSPA8, NUP153
	Activin receptor type II	DDX3X
AMPK	AMPK alpha-1 chain	RPL24
	AMPK alpha-2 chain	RPL24
ATM_ATR	Serine-protein kinase ATR	HSPA8, DDX3X
	Serine-protein kinase ATM	HSPA8, DDX3X
AuroraA	Serine/threonine-protein kinase 6	HIST1H2BL
AuroraC_AuroraB	Serine/threonine-protein kinase 12	DDX3X
CaMKIIalpha_CaMKIIdelta	CaM-kinase II alpha chain	RPL24
CaMKIIbeta_CaMKIIgamma	CaM-kinase II gamma chain	NUP153
CDK2_CDK3	Cell division protein kinase 2	APOB, EIF4A1, NUP153
CDK4_CDK6	Cell division protein kinase 4	HSPA8
	Cell division protein kinase 6	HSPA8
CK2	Casein kinase II, alpha' chain	CTTNBP2, HSPA8, ATP5B, APOB, RPL24, DDX3X, NUP153
	Casein kinase II, alpha chain	CTTNBP2, HSPA8, ATP5B, APOB, RPL24, DDX3X, NUP153
CLK	Dual specificity protein kinase CLK1	DDX3X, HIST1H2BL
	Dual specificity protein kinase CLK2	DDX3X, HIST1H2BL
DMPK	Myotonin-protein kinase	RPL24
EGFR	Receptor tyrosine-protein kinase erbB-1, erbB-2, erbB-3, erbB-4	HSPA8
EphA7_EphA6_EphA4_EphA3_EphA5	Ephrin type-A receptor 4, 7, 3	DDX3X, TPM4
FLT3_CSF1R_Kit	Macrophage colony stimulating factor I receptor	ARPC3, DDX3X
	Mast/stem cell growth factor receptor	ARPC3, DDX3X
GSK3	Glycogen synthase kinase-3 beta	HSPA8, DDX3X, NUP153
	Glycogen synthase kinase-3 alpha	HSPA8, DDX3X, NUP153
InsR	Insulin-like growth factor 1 receptor	HSPA8, DDX3X, NUP153, TPM4
	Insulin receptor	HSPA8, DDX3X, NUP153, TPM4
JNK	Mitogen-activated protein kinase 8	NUP153
	Mitogen-activated protein kinase 10	NUP153
	Mitogen-activated protein kinase 9	NUP153
MAP2K6_MAP2K3_MAP2K4_MAP2K7	Dual specificity mitogen-activated protein kinase kinase 4	HSPA8, DDX3X
	Dual specificity mitogen-activated protein kinase kinase 3	HSPA8, DDX3X
	Dual specificity mitogen-activated protein kinase kinase 6	HSPA8, DDX3X
MAPK3_MAPK1_MAPK7_NLK	Mitogen-activated protein kinase 3	NUP153
	Mitogen-activated protein kinase 1	NUP153
	Mitogen-activated protein kinase 7	NUP153
MAPKAPK	MAP kinase-activated protein kinase 5	RPL24
	MAP kinase-activated protein kinase 2	RPL24
	MAP kinase-activated protein kinase 3	RPL24
Met	Hepatocyte growth factor receptor	HSPA8
NEK1_NEK5_NEK3_NEK4_NEK11_NEK2	Serine/threonine-protein kinase Nek2	HSPA8, DDX3X, NUP153
p38	Mitogen-activated protein kinase 14	NUP153
	Mitogen-activated protein kinase 11	NUP153
	Mitogen-activated protein kinase 13	NUP153
p70S6K	Ribosomal protein S6 kinase 1	DDX3X, NUP153
PAKA	Serine/threonine-protein kinase PAK 1	HSPA8, DDX3X, NUP153
	Serine/threonine-protein kinase PAK 2	HSPA8, DDX3X, NUP153
	Serine/threonine-protein kinase PAK 3	HSPA8, DDX3X, NUP153
PAKB	Serine/threonine-protein kinase PAK 4	DDX3X
	Serine/threonine-protein kinase PAK 7	DDX3X
PDGFR	Beta platelet-derived growth factor receptor	HSPA8
	Alpha platelet-derived growth factor receptor	HSPA8
Pim2	Serine/threonine-protein kinase Pim-2	RPL24, DDX3X
Pim3_Pim1	Proto-oncogene serine/threonine-protein kinase Pim-1	DDX3X
PKA	cAMP-dependent protein kinase, beta-catalytic subunit	NUP153
	cAMP-dependent protein kinase, alpha-catalytic subunit	NUP153
PKC	Protein kinase C, delta type	HIST1H2BL, NUP153
	Protein kinase C, zeta type	HIST1H2BL, NUP153
	Protein kinase C, iota type	HIST1H2BL, NUP153
	Protein kinase C, theta type	HIST1H2BL, NUP153
	Protein kinase C, alpha type	HIST1H2BL, NUP153
	Protein kinase C, gamma type	HIST1H2BL, NUP153
PKD	Protein kinase, D1 type	HSPA8
Tec	Tyrosine-protein kinase ITK/TSK	ARPC3
	Tyrosine-protein kinase BTK	ARPC3
	Tyrosine-protein kinase Tec	ARPC3
TLK	Serine/threonine-protein kinase tousled-like 1	DDX3X, HIST1H2BL

**Figure 4:**
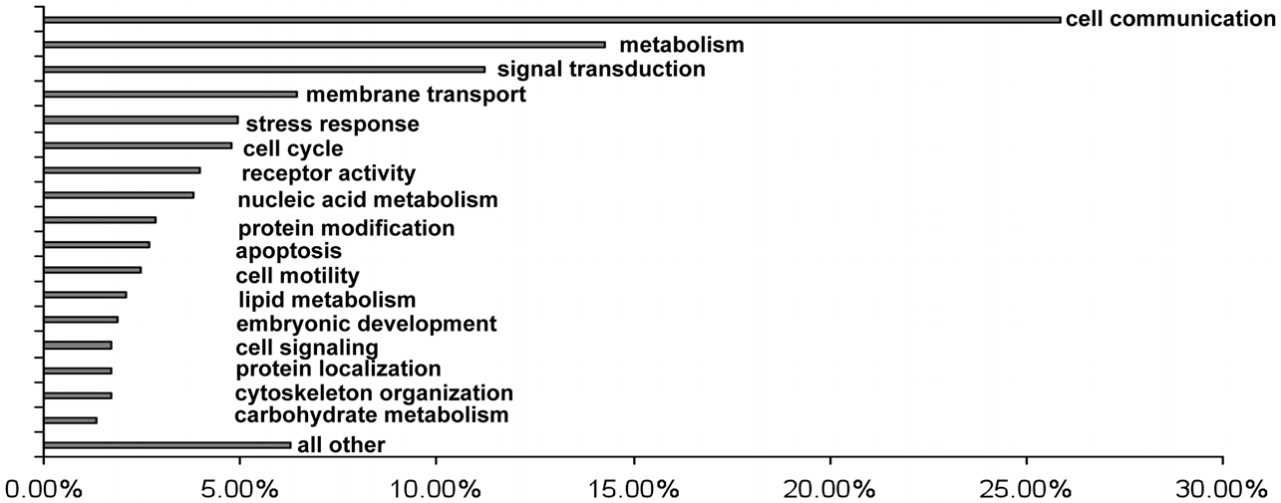
GO categories represented by the 70 kinases. The 70 predicted kinases thought to have the potential to phosphorylate the 11 candidate phosphoproteins were annotated using GOanna and the annotations were categorizied using CateGOrizer which mapped the kinases based on EGAD2GO. The GO terms mapped to 47 EGAD2GO ancestor terms and this graph represents the top 17 definitions and the rest are grouped into the “other” category.

To determine the role these kinases may play in HLA class I cytoskeleton activation the 70 protein kinases were annotated using GOanna [23] and the GO terms were then categorized using CateGOrizer [24]. The top three GO categories represented by the 70 kinases were cell communication (26%), metabolism (14%) and signal transduction (11%) (Fig. 4).

### Validation of protein candidates

**Figure 5:**
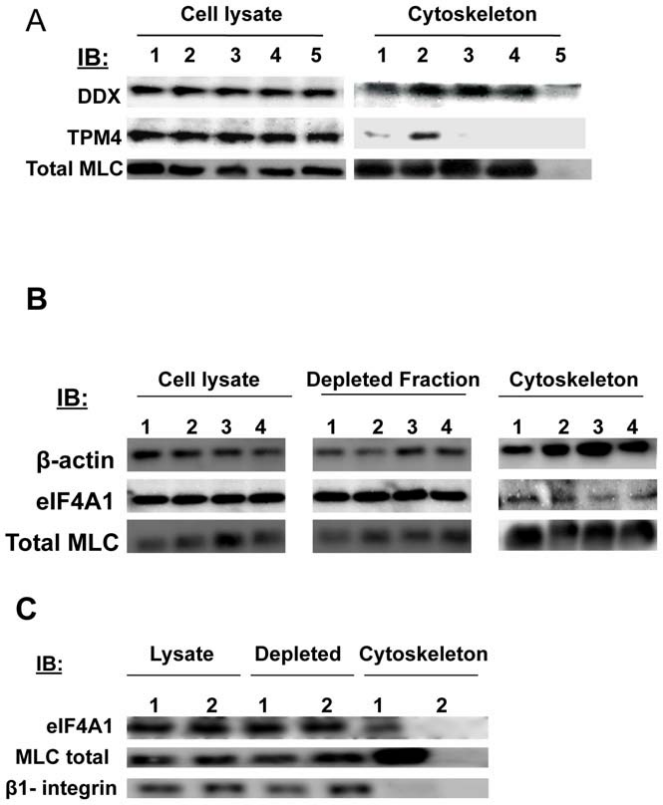
DDX3X, TPM4 and eIF4A1 in the EC cytoskeleton fraction. (A) The EC cytoskeleton was isolated for the 4 treatment groups, mIgG 1 µg/ml (lane 1), HLA class I 1 µg/ml (lane 2), thrombin 1 U/ml (lane 3), bFGF 25 ng/ml (lane 4) and ECs that were treated with cytochalasin D (5 µM, 30 min) (lane 5). The total cell lysate and the cytoskeleton fraction were subjected to SDS-PAGE separation followed by Western blot to examine the presence of MLC, DDX3X and TPM4 proteins. (B) The EC cytoskeleton isolation was isolated for the 4 treatment groups, mIgG 1 µg/ml (lane 1), HLA class I 1 µg/ml (lane 2), thrombin 1 U/ml (lane 3) and bFGF 25 ng/ml (lane 4). The total cell lysates, the depleted fractions and the cytoskeleton isolated fractions were subjected to SDS-PAGE separation followed by Western blot to examine ß-actin, total MLC and eIF4A1 protein levels in the different cell fractions. (C) The cytoskeleton isolation was performed on ECs treated with mIgG 1 µg/ml for 10 min (lane 1) or with cytochalasin D (lane 2) 5 µM for 30 min. The total cell lysates, depleted fractions and cytoskeleton isolated fractions were subjected to SDS-PAGE separation followed by Western blot to examine eIF4A1, total MLC and ß1-integrin in the cell fractions. The results presented in panel A and B are representative of 5 and 3 independent experiments, respectively.

To confirm the presence of the candidate proteins in the cytoskeleton fraction, we performed Western blot experiments with antibodies to 3 of these candidate proteins, DDX3X, TPM4 and eIF4A1 (Table 1). MLC, a well documented component of the actin cytoskeleton, was found in the cytoskeleton fraction of all treatment groups, but absent in the cytoskeleton fractions following treatment with cytochalasin D (Fig. 5A). Similarly, all 4 treatment groups showed DDX3X in the cytoskeleton fraction, but not when the cytoskeleton was disrupted using cytochalasin D. TPM4 was only found in the cytoskeleton fraction of ECs treated with anti-HLA class I antibodies and its association with the cytoskeleton was abrogated by pretreatment with cytochalasin D (Fig. 5A).

Among the list of candidates, eIF4A1 was selected for further characterization because it functions as a downstream target of mammalian target of rapamycin (mTOR) [25] and we previously reported a role for mTOR in class I-mediated cell proliferation [18]. The cytoskeleton isolation was repeated for each treatment group, and various proteins in the cytoskeleton fraction were compared to the total cell lysate and the depleted fraction of the cytoskeleton by Western blot. As expected, the known cytoskeleton components, ß-actin and MLC, were found in the cytoskeleton fraction (Fig. 5B). eIF4A1 was also found in the cytoskeleton fraction of all treatment groups (Fig. 5B). However, eIF4A1 was no longer captured in the cytoskeleton fraction following treatment with cytochalasin D. These data demonstrate that capture of the total cytoskeleton contains eIF4A1 in all 4 of the treatment conditions when examined by Western blot.

**Figure 6:**
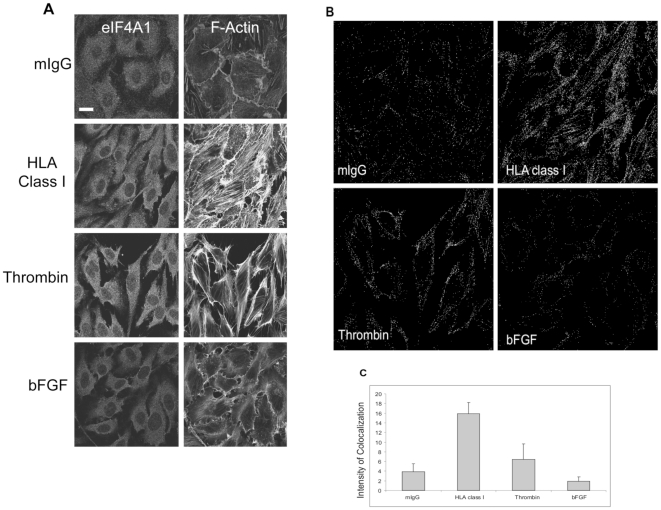
Colocalization of eIF4A1 and F-actin. (A) The localization of eIF4A1 and F-actin in the EC was determined by confocal microscopy. Cell were treated and prepared for staining and microscopy. The scale bar is equal to 10 µm. Data is representative of 4 independent experiments. (B) eIF4A1 and F-actin colocalization was determined by the the ImageJ plugin Colocalization Finder (http://rsbweb.nih.gov/ij/plugins/colocalization-finder.html). Manders' Overlay coefficients were 0.916 (mIgG), 0.926 (HLA class I), 0.942 (thrombin) and 0.921 (bFGF). The data represents 4 independent experiments. (C) Intensities of the colocalization of 3 images per group were determined for each independent experiment (Avg ± SD): mIgG (3.8±1.7), HLA class I (15.9±2.3), thrombin (6.4±3.2) and bFGF (1.9±0.9). The colocalization intensity of eIF4A1 and F-actin in the HLA class I treated group was significantly increased compared to the unstimulated and bFGF groups; mIgG (p = 0.006), thrombin (p = 0.07) and bFGF (p = 0.002) as determined by student t-test.

We next examined HLA class I-induced eIF4A1 interactions with the cytoskeleton by performing cellular colocalization studies. For this, confocal microscopy was performed to determine the degree of colocalization of eIF4A1 with F-actin and paxillin, both of which are known regulators of HLA class I cytoskeleton changes [14]. Figure 6A shows the localization of eIF4A1 and F-actin in the ECs. The colocalization image (Fig. 6B) generated by the ImageJ plugin was used to calculate changes in the level of colocalization (Fig. 6C). The eIF4A1 protein aligned with F-actin stress fibers in both the HLA class I and thrombin stimulated ECs. The degree of colocalization was more prominent in ECs treated with HLA class I antibodies compared to thrombin treated ECs, but this difference did not reach statistical significance (p = 0.07). The colocalization intensity of eIF4A1 and F-actin following HLA class I stimulation was statistically significant when compared to the unstimulated (p = 0.006) and bFGF treated groups (p = 0.002). To assess the specificity of the association between eIF4A1 and the actin cytoskeleton, we repeated this experiment using a second antibody recognizing a different epitope on eIF4A1. We observed a similar pattern of immunfluoresence staining and degree of colocalization, confirming the colocalization of eIF4A1with F-actin stress fibers (Figure S1).

**Figure 7:**
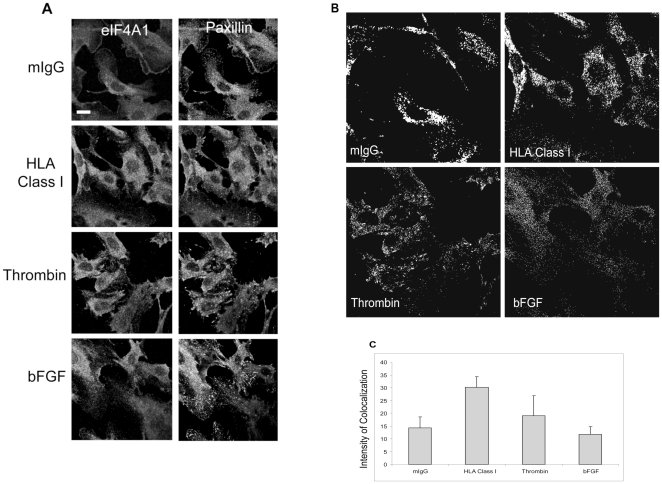
Colocalization of eIF4A1 and Paxillin. (A) The localization of eIF4A1 and paxillin in the EC was determined by confocal microscopy. Cell were treated and prepared for staining and microscopy. The scale bar is equal to 10 µm. Data is representative of 4 independent experiments. (B) eIF4A1 and paxillin colocalization was determined by the ImageJ plugin Colocalization Finder. Mander's Overlap coefficients were 0.977 (mIgG), 0.929 (HLA class I), 0.933 (thrombin) and 0.888 (bFGF). The data represents 4 independent experiments. (C) Intensities of the colocalization of 3 images per group were determined for each independent experiment (Avg ± SD): mIgG (14.3±4.3), HLA class I (30.2±4.1), thrombin (19.1±7.8) and bFGF (11.7±3.0). The colocalization intensity of eIF4A1 and paxillin in the HLA class I treated group was significantly increased compared to the unstimulated and bFGF groups; mIgG (p = 0.01), thrombin (p = 0.09) and bFGF (p = 0.003) as determined by student t-test.

Figure 7A represents the staining pattern and localization of eIF4A1 and paxillin. The colocalization image (Fig. 7B) determined by the ImageJ plugin was used to compare the degree of alignment of these two proteins (Fig. 7C). Similar to the results obtained with F-actin, EC stimulated with HLA class I antibodies and thrombin exhibited a higher degree of colocalization with paxillin than cells treated with bFGF or isotype control IgG. Although the increased alignment of eIF4A1 and paxillin was more prominent after HLA class I ligation compared to thrombin (p = 0.09), the difference was only statistically significant when compared to the mIgG (p = 0.01) and bFGF (p = 0.003) treatment groups. These results indicate increased colocalization of eIF4A1 with F-actin and paxillin following HLA class I stimulation.

## Discussion

The actin cytoskeleton is an important regulator of various cellular functions in ECs including proliferation, migration and permeability [26]. To gain insights into the molecular processes underlying HLA class I-mediated actin stress fiber remodeling we conducted a proteomic analysis of the cytoskeletal fractions of ECs treated with anti-HLA class I antibodies and contrasted these findings to thrombin and bFGF-induced cytoskeletal remodeling. Here we report a novel method to examine the composition of the EC cytoskeleton and reveal unique cytoskeleton proteomes in ECs depending on the agonist used for stimulation. Using annotation tools, a candidate list was created that revealed 12 potential HLA class I induced targets that may play a role in understanding EC cytoskeleton changes.

Among the list of candidates, we identified proteins or related proteins involved in cytoskeleton dynamics. For example, cortactin is a known actin-binding protein and transduces signaling to the cytoskeleton [27], [28]. It also has the ability to cross-link F-actin, which is regulated by Src-kinase mediated activation [29]. Additionally, the actin-related protein 2/3 (Arp2/3) complex is essential in the regulation of actin polymerization and localizes to sites of dynamic actin [30]. To tie these processes together one group reported that cortactin colocalizes with both dynamic actin and the Arp2/3 complex [31]. HLA class I ligation on the surface of ECs stimulates the formation of actin stress fibers and the activation of Src family kinases [14]. Thus, it is plausible that HLA class I-mediated Src kinase phosphorylation may regulate cortactin binding protein 2 and Arp 2/3 complex subunit 3 in the formation of stress fibers following class I ligation.

In the past, we established that HLA class I signaling cascades involve various phosphorylation events and have explored several key proteins responsible for these events [14], [32]. The current data add to our understanding of these phosphorylation events as we explored the kinases that have the potential to phosphorylate the candidate proteins. Among the kinase predictions, cyclin dependent kinase 2 (CDK2 has the potential to phosphorylate 3 of the candidate proteins. CDK2 has already been established as a regulator of HLA class I signal transduction. HLA class I ligation on the surface of ECs leads to the inactivation of retinoblastoma protein (Rb) and this happens through CDK2 [33]. Rb is negative regulator of cell cycle progression [34]. Activation of CDK2 relieves the inhibition put forth by Rb and this permits the ECs to pass the G_1_ checkpoint and proliferate [33]. The current study elucidated several candidate targets of CDK2, which may contribute to a better understanding of the molecular processes involved in HLA class I-mediated signal transduction leading to cell proliferation.

Another kinase family predicted to be involved in the phosphorylation of the candidate proteins is 70-kDa S6 protein kinase (p70S6k) family, where the specific kinase was ribosomal protein S6 kinase 1 (S6RP). HLA class I ligation leads to the phosphorylation of p70S6k and S6RP, which are downstream of mTOR complex 1 [18]. It has yet to be determined which S6RP downstream targets are important in HLA class I signaling. The current findings suggest that S6RP may phosphorylate two of the candidate proteins, ATP-dependent RNA helicase DDX3X and nuclear pore complex protein Nup153. DDX3X was confirmed by Western blot to be associated with the isolated cytoskeleton fraction following HLA class I ligation on ECs. DDX3X interacts with TANK binding kinase 1 to activate the interferon (IFN) promoter. When DDX3X protein levels are reduced using RNAi, type I IFN production is diminished [35]. Cytokine production is associated with allograft rejection [36] and *in vitro*, cytokines are known to synergize with HLA class I antibodies to enhance signal transduction [37]. Little is known about the regulation of HLA class I induced cytokine production and DDX3X may be a potential target in controlling cytokine induction. Recent findings suggest that Nup153 is crucial for nucleoskeleton and cytoskeleton architecture maintenance and is necessary for cell cycle progression and cell migration. In addition, when Nup 153 is knocked down by RNAi, there is prominent cytoskeleton rearrangement that hinders cell migration in human breast carcinoma cells [38]. The integrity of the cytoskeleton is key to HLA class I signaling [14], [15] and given the role of Nup153 it seems that this protein would be worth investigating.

TPM4 was identified as a candidate protein in the HLA class I treated group by mass spectrometry and confirmed by Western blot to be present only in the cytoskeleton fraction of HLA class I stimulated ECs. Tropomyosins are among the most abundant cytoskeletal proteins in ECs [39]. By mass spectrometry, tropomyosin-1 was identified as an oxidative-stress-sensitive phosphoprotein in ECs [40]. H2O2 induced the activation of DAP kinase, downstream of ERK. DAP kinase promoted the phosphorylation of tropomyosin-1, which was essential for H2O2 induced formation of actin stress fibers in ECs [41]. HLA class I ligation leads to ERK activation [18] and thus, it would be valuable to determine if TPM4 functions downstream of ERK as regulator of HLA class I induced cytoskeleton remodeling.

The eIF4A1 protein, functions downstream of mTOR complex 1, which has been shown to phosphorylate 4E-BP1 following class I ligation [18]. 4E-BP1 is bound to eIF4E and when 4E-BP1 becomes phosphorylated eIF4E is released and recruited to eIF4F, which includes both eIF4A1 and eIF4G. This complex promotes translation and cell proliferation [25]. The link between translational machinery and the cytoskeleton was postulated some time ago [42]. Continued efforts to make this connection showed that when the soluble fraction of a cell lysate was compared to the cytoskeleton fraction, certain initiation factors where enriched in the cytoskeleton and one of those factors was eIF4A1 [43]. It has yet to be determined how the cytoskeleton could regulate and organize translation.

Although eIF4A1 and DDX3X were found by mass spectrometry to be exclusively in the HLA class I treated EC, surprisingly these proteins were found at approximately equal levels in all of the treatment groups following cytoskeleton isolation and Western blotting. The most likely explanation for the detection of eIF4A1 and DDX3X by mass spectrometry only in the class I treated group is the difference in the sensitivity of these assays. In the ECs treated with bFGF or thrombin, other more abundant cytoskeletal proteins may have been present which reduced the relative amount of DDX3X and eIF4A1, precluding their detection by mass spectrometry. Indeed, even when highly sensitive mass spectrometers are used to analyze complex biological samples and bodily fluids, high-abundance proteins obscure the detection of lower-abundance proteins [44].

An alternative explanation for these discrepant findings is that different protein:protein interactions in the treatment groups influence the ability to detect a protein by mass spectrometry. For example, the function of eIF4A1 has little to do with its protein level and more to do with whether or not it is being inhibited [45]. Programmed Cell Death 4 (PDCD4) is a tumor suppressor known to bind eIF4A1, which inhibits translation initiation and proliferation [46]. Detection of eIF4A1 in the cytoskeleton of all treatment groups by Western blot would not be hindered if it is bound to PDCD4, but detection by mass spectrometry could be masked.

We found that treatment with HLA class I antibodies or thrombin stimulated varying degrees of colocalization between eIF4A1 and F-actin and paxillin suggesting that eIF4A1 may interact with specific compartments of the cytoskeleton in a unique manner. Consistent with this concept, recent studies by our laboratory identified two different signaling pathways leading to MLC phosphorylation and stress fiber formation in ECs, depending upon the nature of the stimulus (ME Ziegler, unpublished). Stimulation with thrombin at 1 U/ml induced a robust increase in the intracellular Ca^2+^ concentration, increased phosphorylation of MLC and promoted stress fiber formation via MLCK and ROK in an ERK-independent manner. In contrast, stimulation of ECs with a low dose of thrombin (1 mU/ml) or HLA class I antibodies did not promote any detectable change in intracellular Ca^2+^ concentration, but induced MLC phosphorylation and stress fiber assembly via MLCK and ROK in an ERK1/2-dependent manner. HLA class I ligation requires the recruitment of integrin ß4 in order to activate proliferation and migration [47]. In carcinoma migration, α6ß4 integrin functions via its ability to promote and stabilize F-actin [48]. Additionally, eukaryotic initiation factors in migrating cells localize with focal adhesions, which contain actin and this up-regulation of localized translation is thought to be a result of integrin engagement [49], [50]. These data suggest that the increased colocalization of eIF4A1 with F-actin and paxillin following class I ligation may be in response to the HLA class I molecule partnering with integrin ß4 to elicit intracellular signals. Although the colocalization data supports a molecular interaction between eIF4A1 and F-actin and paxillin, additional experiments are required to definitively prove a physical interaction between these proteins. eIF4A1 was suggested to be a potential target for developing new anti-cancer and anti-inflammatory drugs given evidence of cross-talk between translation and the inflammatory response [51]. Our findings suggest that eIF4A1 may also be a potential therapeutic target of HLA class I induced antibody-mediated rejection.

An important functional consequence of HLA class I ligation on ECs is stimulation of cell proliferation, which we previously reported to occur in subconfluent ECs [15], [52]. To gain knowledge on the cytoskeleton changes involved in class I-mediated cell proliferation, we used subconfluent ECs to conduct these experiments. However, the EC density is an important factor that influences cellular responses. Confluent ECs regulate thrombosis, inflammation, vascular cell proliferation and matrix remodeling, whereas subconfluent ECs promote these events [53]. Cell morphology, expression of cell surface molecules and behavior of the ECs differ in subconfluent versus confluent ECs [54]. Additionally, the EC response to an agonist varies depending on cell density [55], [56]. A quiescent EC monolayer is more similar to the *in vivo* setting in appearance and differentiated properties [57], [58]. We have yet to explore the cytoskeletal proteome of a confluent monolayer of ECs in response to HLA class I ligation and postulate that a confluent monolayer may respond differently and utilize distinct signaling pathways.

A key question is how signal transduction is orchestrated through these molecular interactions to stimulate actin cytoskeletal remodeling. Our previous publications and current data are consistent with a model whereby molecular aggregation of HLA class I molecules with antibodies leads to recruitment of integrin ß4 and the subsequent activation of intracellular signals that increase Rho-GTP activity, induce phosphorylation of ROK and trigger the assembly and phosphorylation of FAK, Src and paxillin at the focal adhesions to stimulate actin reorganization [13], [14], [15], [47]. The new candidate proteins identified in this study may further contribute to this model. Candidates such as TPM4, eIF4A1, DDX3X, cortactin binding protein 2 and Arp2/3 may be recruited to the focal adhesions to regulate cell proliferation and survival. Similar signaling cascades have been described following antibody cross-linking of ICAM-1 on ECs. ICAM-1 ligation induced cytoskeleton changes, which included increased intracellular calcium, protein kinase C activation, phosphorylation of cortactin and other actin-binding proteins by Src, activation of RhoA GTPase, and subsequent rearrangements of the actin [59], [60], [61], [62], [63].

In conclusion, these studies provide new information that can be applied to the exploration of known pathways. Given that phosphoprotein signal transduction is essential to HLA class I EC activation, not only are the proteins relevant, but also their corresponding kinases. Thus, validation of these proteins and examination of their activation state will be important in future studies. Overall these studies may reveal more specific targets in understanding the mechanisms of HLA class I induced antibody-mediated rejection. Additionally, these methods can be applied to other cell types and agonists as an effort to understand the role of cytoskeleton changes in many pathways.

## Supporting Information

Figure S1 **Validation of eIF4A1 localization.** The localization of eIF4A1 and F-actin by confocal microscopy in ECs treated with (A) mIgG (1 µg/ml), (B) and HLA class I antibody (1 µg/ml) was performed using the eIF4A1 antibody used in Figure 6 (eIF4A1 Ab 1) and an additional antibody, which recognizes a different epitope of eIF4A1 (eIF4A1 Ab 2). A high-resolution image of an individual EC is shown for each antibody. The scale bar is equal to 1 µm. (C) The localization of eIF4A1 and F-actin for the 2 eIF4A1 antibodies showing multiple ECs in each field. The scale bar is equal to 10 µm. (D) The colocalization of eIF4A1 and F-actin was determined by the ImageJ plugin, Colocalization Finder. The Manders' Overlay coefficients for the experiment using eIF4A1 Ab 1 were 0.934 (mIgG) and 0.918 (HLA class I). Intensities of the colocalization of 3 images per group were determined (Avg ± SD): mIgG (4.2±0.35) and HLA class I (17.8±2.0). The colocalization intensity of eIF4A1 Ab 1 and F-actin in the HLA class I treated group was significantly increased compared to the unstimulated p = 0.004 as determined by student t-test. The Manders' Overlay coefficients for the experiment using eIF4A1 Ab 2 were 0.914 (mIgG) and 0.933 (HLA class I). Intensities of the colocalization of 3 images per group were determined (Avg ± SD): mIgG (5.3±0.85) and HLA class I (19.1±3.2). The colocalization intensity of the eIF4A1 Ab 2 and F-actin in the HLA class I treated group was significantly increased compared to the unstimulated p = 0.01 as determined by student t-test.
(PPTX)

Table S1 **Identity of Proteins in the Cytoskeleton Preparations of Each Treatment Group.** To identify the proteins in the cytoskeleton isolation preparations, nLC-MS/MS was performed on the peptides and Mascot searches were carried out. A total of 128 cytoskeleton-associated proteins were identified in unstimulated ECs, 126 in HLA class I stimulated ECs, 67 in thrombin treated ECs and 88 in bFGF treated ECs.
(DOC)
