# Supplementary material for: Characterization of the Endothelial Cell Cytoskeleton following HLA Class I Ligation
Source: PLoS One. 2012 Jan 11;7(1):e29472. doi: 10.1371/journal.pone.0029472 (PMC3256144; doi:10.1371/journal.pone.0029472)
Supplement: Figure S1 — Validation of eIF4A1 localization. The localization of eIF4A1 and F-actin by confocal microscopy in ECs treated with (A) mIgG (1 µg/ml), (B) and HLA class I antibody (1 µg/ml) was performed using the eIF4A1 antibody used in Figure 6 (eIF4A1 Ab 1) and an additional antibody, which recognizes a different epitope of eIF4A1 (eIF4A1 Ab 2). A high-resolution image of an individual EC is shown for each antibody. The scale bar is equal to 1 µm. (C) The localization of eIF4A1 and F-actin for the 2 eIF4A1 antibodies showing multiple ECs in each field. The scale bar is equal to 10 µm. (D) The colocalization of eIF4A1 and F-actin was determined by the ImageJ plugin, Colocalization Finder. The Manders' Overlay coefficients for the experiment using eIF4A1 Ab 1 were 0.934 (mIgG) and 0.918 (HLA class I). Intensities of the colocalization of 3 images per group were determined (Avg ± SD): mIgG (4.2±0.35) and HLA class I (17.8±2.0). The colocalization intensity of eIF4A1 Ab 1 and F-actin in the HLA class I treated group was significantly increased compared to the unstimulated p = 0.004 as determined by student t-test. The Manders' Overlay coefficients for the experiment using eIF4A1 Ab 2 were 0.914 (mIgG) and 0.933 (HLA class I). Intensities of the colocalization of 3 images per group were determined (Avg ± SD): mIgG (5.3±0.85) and HLA class I (19.1±3.2). The colocalization intensity of the eIF4A1 Ab 2 and F-actin in the HLA class I treated group was significantly increased compared to the unstimulated p = 0.01 as determined by student t-test. (PPTX) [file pone.0029472.s001.pptx]

## Slide 1
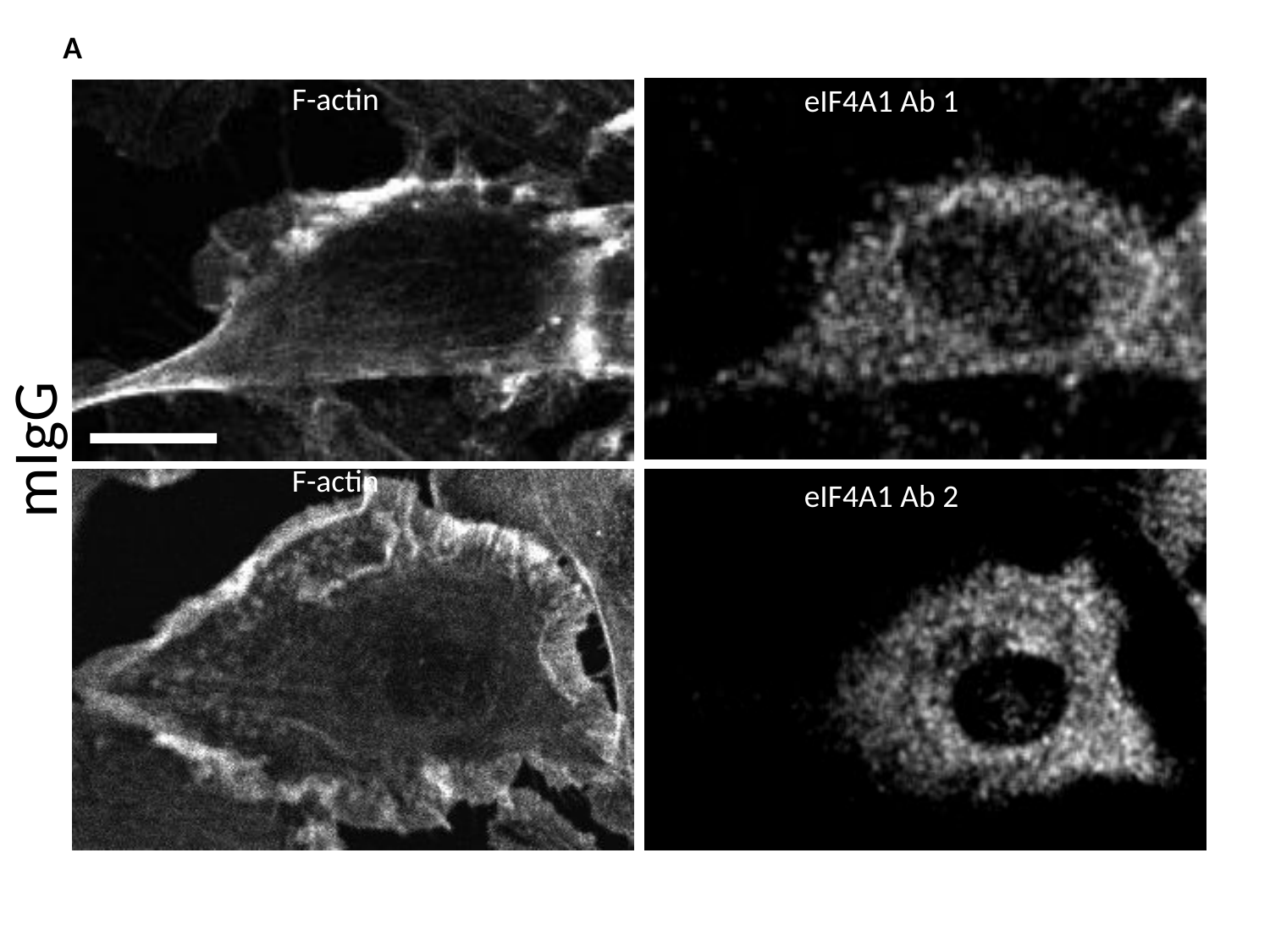

A
F-actin
eIF4A1 Ab 1
mIgG
F-actin
eIF4A1 Ab 2

## Slide 2
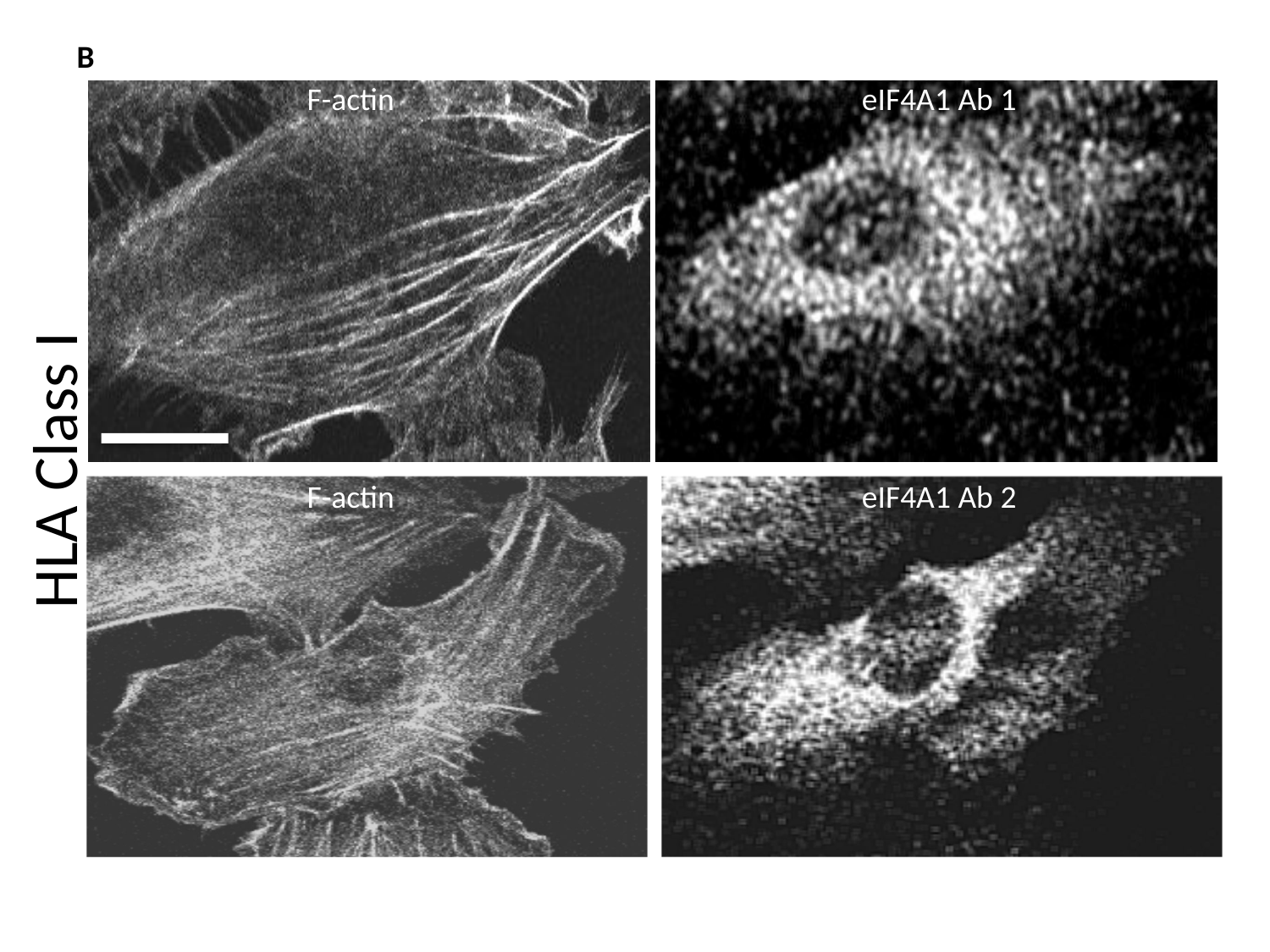

B
F-actin
eIF4A1 Ab 1
HLA Class I
F-actin
eIF4A1 Ab 2

## Slide 3
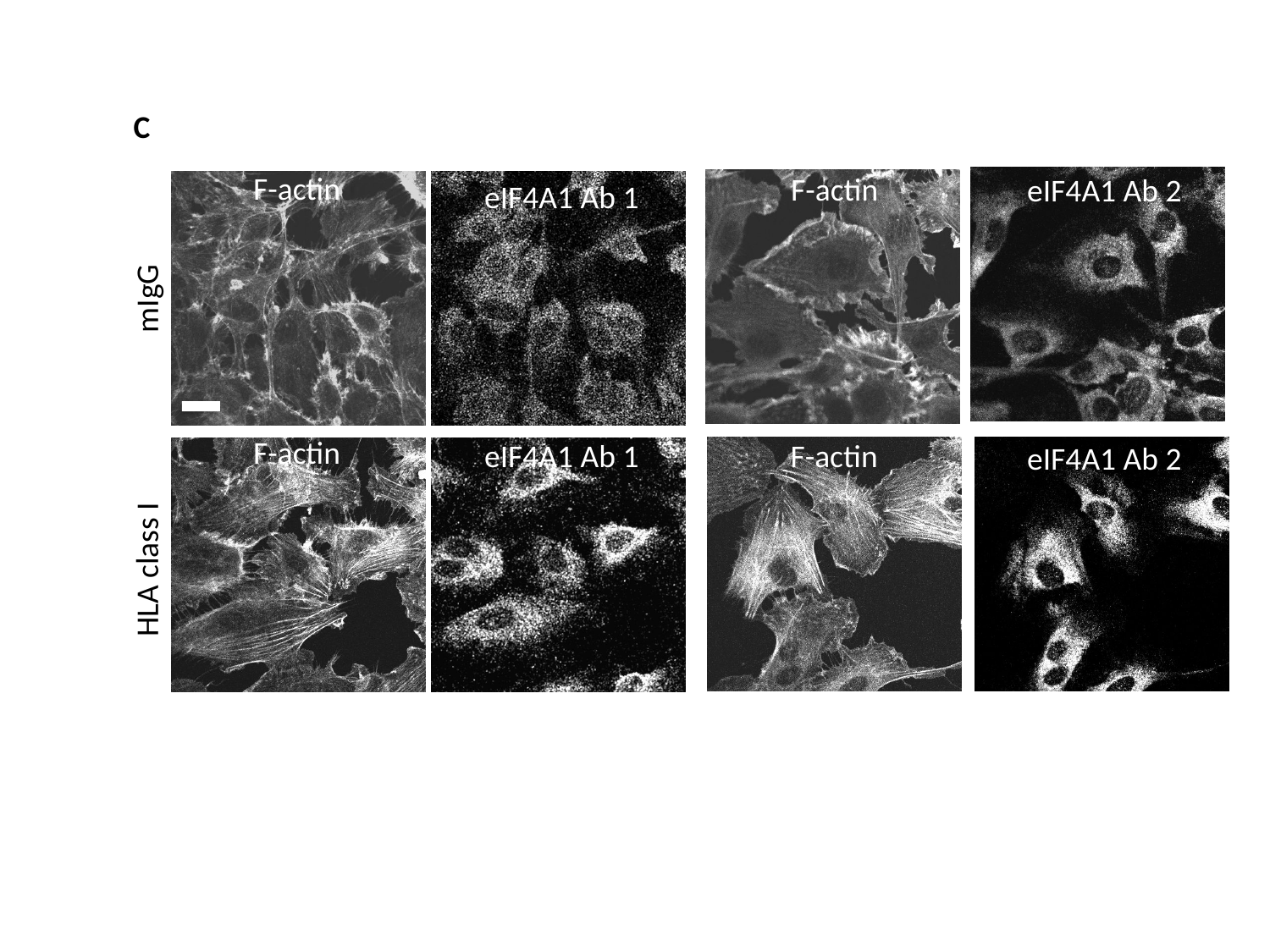

C
F-actin
F-actin
eIF4A1 Ab 2
eIF4A1 Ab 1
mIgG
F-actin
eIF4A1 Ab 1
F-actin
eIF4A1 Ab 2
HLA class I

## Slide 4
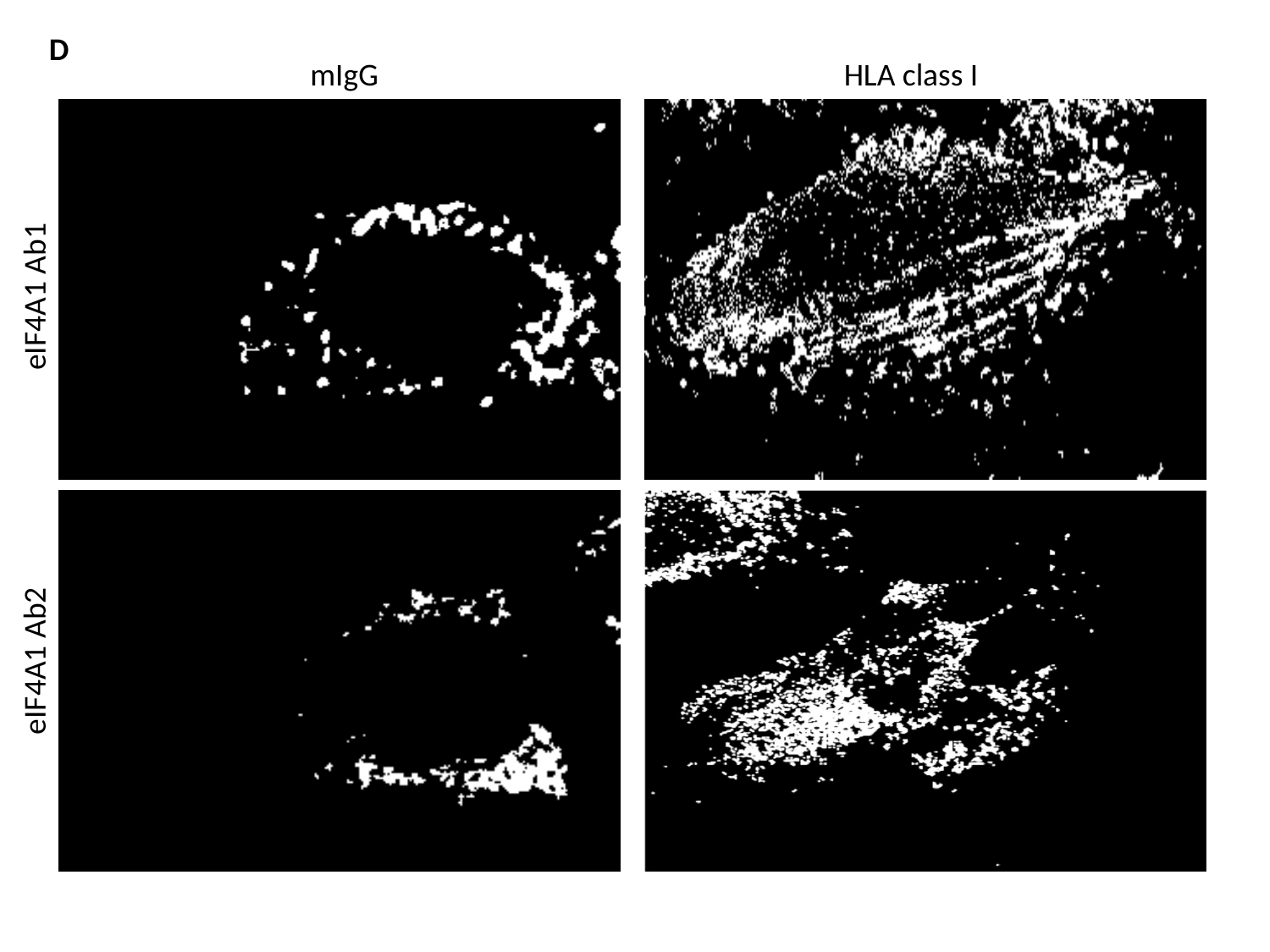

D
mIgG
HLA class I
eIF4A1 Ab1
eIF4A1 Ab2
