# Supplementary material for: Characterization of the Endothelial Cell Cytoskeleton following HLA Class I Ligation
Source: PLoS One. 2012 Jan 11;7(1):e29472. doi: 10.1371/journal.pone.0029472 (PMC3256144; doi:10.1371/journal.pone.0029472)
Supplement: Table S1 — Identity of Proteins in the Cytoskeleton Preparations of Each Treatment Group. To identify the proteins in the cytoskeleton isolation preparations, nLC-MS/MS was performed on the peptides and Mascot searches were carried out. A total of 128 cytoskeleton-associated proteins were identified in unstimulated ECs, 126 in HLA class I stimulated ECs, 67 in thrombin treated ECs and 88 in bFGF treated ECs. (DOC) [file pone.0029472.s002.doc]

**Supplemental Table 1**. Identity of Proteins in the Cytoskeleton Preparations

of Each Treatment Group

|  |  | **Mascot Score** | | | |
| --- | --- | --- | --- | --- | --- |
| **Accession** | **Protein Name** | **mIgG** | **HLA class I** | **Thrombin** | **bFGF** |
| A4FTV3 | Zinc finger protein 185 |  | 60 |  | 77 |
| A4QPB0 | IQGAP1 |  |  | 92 | 73 |
| O00571 | ATP-dependent RNA helicase DDX3X |  | 58 |  |  |
| O14556 | GAPDHS |  | 98 |  |  |
| O14924 | Regulator of G-protein signaling 12 | 58 |  |  | 46 |
| O14950 | Myosin regulatory light chain 12B | 125 | 213 |  |  |
| O15072 | ADAMTS-3 | 40 |  |  |  |
| O15145 | Actin-related protein 2/3 complex subunit 3 |  | 60 |  |  |
| O15212 | Prefoldin subunit 6 | 55 |  |  |  |
| O15231 | Zinc finger protein 185 | 49 |  |  |  |
| O15260 | Surfeit locus protein 4 |  | 42 |  |  |
| O43707 | Alpha-actinin-4 |  |  |  | 331 |
| O60331 | Phosphatidylinositol-4-phosphate 5-kinase | 60 |  |  |  |
| O60447 | EVI-5 |  | 42 |  |  |
| O75369 | Filamin-B | 487 | 368 | 303 | 571 |
| O75526 | RNA-binding motif protein, X-linked-like-2 | 55 | 80 |  |  |
| O76068 | Peripheral-type benzodiazepine receptor |  |  | 43 |  |
| O95049 | Tight junction protein ZO-3 |  |  | 39 |  |
| O95074 | Bax epsilon | 60 | 70 |  |  |
| O95759 | TBC1 domain family member 8 |  | 87 |  |  |
| O95817 | BAG family molecular chaperone regulator 3 |  | 55 |  |  |
| O95831 | Apoptosis-inducing factor 1, mitochondrial |  | 50 |  |  |
| P00338 | L-lactate dehydrogenase A chain |  |  | 103 | 136 |
| P02100 | Hemoglobin subunit epsilon |  | 45 |  |  |
| P02545 | Lamin-A/C | 288 | 192 | 332 | 141 |
| P02751 | Fibronectin | 44 |  |  |  |
| P04114 | Apolipoprotein B-100 |  | 42 |  |  |
| P04275 | von Willebrand factor | 169 | 289 | 82 | 115 |
| P04350 | Tubulin beta-4 chain |  |  | 364 | 411 |
| P04406 | GAPDH | 50 | 150 | 106 | 186 |
| P04792 | Heat shock protein beta-1 | 44 | 145 |  |  |
| P04908 | Histone H2A type 1-B/E | 773 | 2506 |  |  |
| P05141 | ADP/ATP translocase 2 |  |  | 46 | 69 |
| P05976 | Myosin light chain 1/3 | 89 |  |  |  |
| P06133 | UDP-glucuronosyltransferase 2B4 | 41 |  |  |  |
| P06576 | ATP synthase subunit beta, mitochondrial |  | 40 |  |  |
| P06733 | Alpha-enolase | 59 |  |  |  |
| P06748 | Nucleophosmin |  | 39 | 90 | 92 |
| P07195 | L-lactate dehydrogenase B chain |  |  | 54 | 54 |
| P07355 | Annexin A2 | 57 |  |  | 47 |
| P07437 | Tubulin beta chain | 164 | 140 | 557 | 457 |
| P07602 | Proactivator polypeptide |  |  |  | 65 |
| P07900 | Heat shock protein HSP 90-alpha |  |  | 47 | 51 |
| P07910 | hnRNP C1/C2 | 40 |  |  |  |
| P08238 | Heat shock protein HSP 90-beta |  |  | 192 | 236 |
| P08590 | Myosin light chain 3 |  |  |  | 150 |
| P08670 | Vimentin | 14886 | 15196 | 7610 | 15269 |
| P09651 | hnRNP A1 | 362 |  | 104 | 97 |

|  |  | **Mascot Score** | | | |
| --- | --- | --- | --- | --- | --- |
| **Accession** | **Protein Name** | **mIgG** | **HLA class I** | **Thrombin** | **bFGF** |
| P0C0S8 | Histone H2A type 1 | 58 | 85 |  |  |
| P11142 | Heat shock cognate 71 kDa protein |  | 60 |  |  |
| P12036 | Neurofilament heavy polypeptide |  |  |  | 54 |
| P12814 | Alpha-actinin-1 | 141 | 234 |  | 284 |
| P13010 | ATP-dependent DNA helicase 2 subunit 2 |  |  | 59 |  |
| P13639 | Elongation factor 2 | 48 |  |  |  |
| P13987 | CD59 glycoprotein |  | 189 |  |  |
| P14618 | Pyruvate kinase isozymes M1/M2 | 126 |  |  |  |
| P14649 | Myosin light chain 6B | 292 | 95 |  |  |
| P15144 | Aminopeptidase N |  | 76 |  | 113 |
| P15880 | 40S ribosomal protein S2 |  | 55 |  |  |
| P16157 | Ankyrin-1 | 44 |  |  |  |
| P16333 | Cytoplasmic protein NCK1 | 53 | 78 |  |  |
| P16402 | Histone H1.3 |  |  |  | 165 |
| P16989 | DNA-binding protein A |  | 55 |  |  |
| P17096 | HMG-I(Y) | 67 | 91 | 40 |  |
| P17655 | Calpain-2 catalytic subunit |  |  |  | 66 |
| P17661 | Desmin |  | 110 | 247 | 110 |
| P18621 | 60S ribosomal protein L17 | 48 |  |  |  |
| P19105 | Myosin regulatory light chain 12A |  |  |  | 369 |
| P19971 | Thymidine phosphorylase | 56 | 44 |  |  |
| P21333 | Filamin-A | 197 | 206 | 195 | 239 |
| P21980 | Protein-glutamine gamma-glutamyltransferase 2 | 45 | 90 |  |  |
| P22492 | Histone H1t |  |  | 158 |  |
| P22626 | hnRNP A2/B1 | 218 | 669 | 271 | 217 |
| P23246 | Splicing factor, proline- and glutamine-rich | 43 | 42 |  |  |
| P23396 | 40S ribosomal protein S3 |  | 59 |  |  |
| P23458 | Tyrosine-protein kinase JAK1 |  | 60 |  |  |
| P23528 | Cofilin-1 |  |  | 120 |  |
| P23528 | Cofilin-1 |  |  |  | 174 |
| P26599 | Polypyrimidine tract-binding protein 1 |  |  |  | 73 |
| P31152 | Mitogen-activated protein kinase 4 | 79 |  |  |  |
| P31942 | hnRNP H3 | 60 | 70 |  |  |
| P31943 | hnRNP H | 125 |  |  |  |
| P35249 | Replication factor C subunit 4 |  | 40 |  |  |
| P35579 | Myosin-9 | 2117 | 2354 | 1776 | 190 |
| P35580 | Myosin-10 |  |  | 101 |  |
| P36578 | 60S ribosomal protein L4 | 76 |  |  | 42 |
| P36873 | PP-1G | 80 | 114 | 64 | 62 |
| P37802 | Transgelin-2 |  | 59 |  |  |
| P39019 | 40S ribosomal protein S19 |  |  | 53 |  |
| P41219 | Peripherin | 508 | 139 |  |  |
| P42345 | Serine/threonine-protein kinase mTOR | 53 | 53 |  |  |
| P43243 | Matrin-3 | 121 | 42 | 71 |  |
| P46782 | 40S ribosomal protein S5 |  | 78 |  |  |
| P47756 | F-actin-capping protein subunit beta | 81 | 44 |  | 73 |
| P48681 | Nestin | 360 | 498 | 192 | 300 |
| P48960 | CD97 antigen |  | 40 |  |  |

|  |  | **Mascot Score** | | | |
| --- | --- | --- | --- | --- | --- |
| **Accession** | **Protein Name** | **mIgG** | **HLA class I** | **Thrombin** | **bFGF** |
| P49790 | Nuclear pore complex protein Nup153 |  | 52 |  |  |
| P49815 | Tuberin | 45 |  |  |  |
| P50454 | Serpin H1 |  |  | 57 | 69 |
| P51991 | hnRNP A3 | 152 | 315 | 68 | 44 |
| P52209 | 6-phosphogluconate dehydrogenase |  |  | 121 | 180 |
| P52272 | hnRNP U | 87 |  |  |  |
| P52272 | hnRNP M |  | 92 |  |  |
| P52597 | hnRNP F | 76 |  | 51 | 115 |
| P54098 | DNA polymerase subunit gamma-1 | 48 | 40 |  |  |
| P54652 | Heat shock-related 70 kDa protein 2 | 79 |  |  |  |
| P55036 | 26S proteasome regulatory subunit S5A |  | 39 |  |  |
| P55060 | Exportin-2 | 38 | 38 |  |  |
| P57088 | Transmembrane protein 33 |  | 50 |  |  |
| P60606 | Cortexin-1 |  | 64 |  |  |
| P60660 | Myosin light polypeptide 6 | 241 | 164 | 227 | 279 |
| P60709 | Actin, cytoplasmic 1 | 990 | 1689 | 647 | 4274 |
| P60842 | Eukaryotic initiation factor 4A-I |  | 59 |  |  |
| P61158 | Actin-related protein 3 | 50 |  |  |  |
| P61978 | hnRNP K | 153 | 154 | 166 | 134 |
| P62081 | 40S ribosomal protein S7 |  |  |  | 69 |
| P62136 | PP-1A |  |  |  | 54 |
| P62241 | 40S ribosomal protein S8 |  | 70 |  |  |
| P62263 | 40S ribosomal protein S14 |  |  | 44 |  |
| P62269 | 40S ribosomal protein S18 |  | 55 |  |  |
| P62314 | Small nuclear ribonucleoprotein Sm D1 |  |  | 42 | 52 |
| P62701 | 40S ribosomal protein S4, X isoform | 54 |  |  | 54 |
| P62736 | Actin, aortic smooth muscle | 70 | 80 |  |  |
| P62753 | 40S ribosomal protein S6 | 45 |  |  |  |
| P62805 | Histone H4 | 282 | 223 | 322 | 186 |
| P62807 | Histone H2B type 1-C/E/F/G/I | 78 | 4845 | 675 | 427 |
| P62829 | 60S ribosomal protein L23 | 44 |  |  |  |
| P62913 | 60S ribosomal protein L11 |  | 77 |  |  |
| P62988 | Ubiquitin |  | 38 |  | 53 |
| P63261 | Actin, cytoplasmic 2 | 50 | 205 |  |  |
| P67809 | Nuclease-sensitive element-binding protein 1 | 56 | 155 |  | 121 |
| P67936 | Tropomyosin alpha-4 chain |  | 82 |  |  |
| P68104 | Elongation factor 1-alpha 1 |  |  | 154 | 171 |
| P68133 | Actin, alpha skeletal muscle |  |  | 85 | 436 |
| P68366 | Tubulin alpha-4A chain | 60 | 145 |  |  |
| P68371 | Tubulin beta-2C chain | 70 | 150 |  |  |
| P68431 | Histone H3.1 |  |  | 285 | 227 |
| P69905 | Hemoglobin subunit alpha |  | 80 |  |  |
| P78332 | RNA-binding protein 6 |  | 41 |  |  |
| P82675 | 28S ribosomal protein S5, mitochondrial |  |  | 83 | 63 |
| P83731 | 60S ribosomal protein L24 |  | 60 |  |  |
| P84098 | 60S ribosomal protein L19 | 40 |  |  |  |
| P84103 | Splicing factor, arginine/serine-rich 3 |  |  | 38 |  |
| P84243 | Histone H3 | 567 | 922 | 226 | 39 |

|  |  | **Mascot Score** | | | |
| --- | --- | --- | --- | --- | --- |
| **Accession** | **Protein Name** | **mIgG** | **HLA class I** | **Thrombin** | **bFGF** |
| P98160 | HSPG2 | 45 |  |  |  |
| Q00610 | Clathrin heavy chain 1 |  |  |  | 88 |
| Q00839 | hnRNP M | 106 | 54 | 39 |  |
| Q01082 | Spectrin beta chain |  |  |  | 51 |
| Q02846 | Retinal guanylyl cyclase 1 | 45 |  |  |  |
| Q02878 | 60S ribosomal protein L6 | 71 |  | 112 | 66 |
| Q04446 | 1,4-alpha-glucan-branching enzyme | 41 |  |  |  |
| Q05469 | Hormone-sensitive lipase | 41 | 40 |  |  |
| Q05639 | Elongation factor 1-alpha 2 | 46 |  |  | 52 |
| Q05BW3 | NES protein | 56 |  |  |  |
| Q07020 | 60S ribosomal protein L18 |  | 77 |  |  |
| Q07021 | Glycoprotein gC1qBP |  |  |  | 40 |
| Q08211 | ATP-dependent RNA helicase A | 41 |  |  |  |
| Q09666 | Neuroblast differentiation-associated protein |  |  | 48 | 50 |
| Q12769 | Nuclear pore complex protein Nup160 |  | 85 |  | 84 |
| Q13148 | TAR DNA-binding protein 43 | 55 | 71 |  |  |
| Q13451 | Peptidyl-prolyl cis-trans isomerase FKBP5 | 52 |  |  |  |
| Q13625 | Apoptosis-stimulating of p53 protein 2 |  | 43 |  |  |
| Q13727 | AHNAK-related protein |  |  | 48 | 50 |
| Q13813 | Spectrin alpha chain |  | 44 | 49 |  |
| Q14315 | Filamin-C |  |  | 67 | 59 |
| Q14839 | CHD-4 | 47 |  |  |  |
| Q14C60 | C21orf29 protein |  | 46 |  |  |
| Q14D04 | VEPH1 | 48 |  |  |  |
| Q15149 | Plectin-1 | 269 | 148 | 56 | 183 |
| Q15233 | NonO protein |  |  |  | 46 |
| Q15293 | Reticulocalbin-1 |  |  | 62 | 60 |
| Q15811 | Intersectin-1 |  | 54 |  |  |
| Q16352 | Alpha-internexin | 51 | 70 |  |  |
| Q16555 | Dihydropyrimidinase-related protein 2 |  | 70 |  |  |
| Q16643 | Drebrin | 85 | 88 |  | 64 |
| Q16778 | Histone H2B type 2-E | 76 | 89 |  |  |
| Q2VIN3 | RBM1 | 40 |  |  |  |
| Q3KQU3 | MAP7 domain-containing protein 1 | 45 |  |  |  |
| Q3KR37 | GRAM domain-containing protein 1B |  | 41 |  |  |
| Q3ZCM7 | Tubulin beta-8 chain |  |  |  | 154 |
| Q4TT55 | Chromosome 16 open reading frame 35 |  | 45 |  |  |
| Q562R1 | Beta-actin-like protein 2 | 364 | 156 | 200 | 325 |
| Q58FF6 | Putative heat shock protein HSP 90-beta 4 |  |  | 58 | 57 |
| Q58FF8 | Putative heat shock protein HSP 90-beta 2 |  |  |  | 64 |
| Q5T4Y8 | MAGI1 |  | 40 |  |  |
| Q60FE2 | Non-muscle myosin heavy polypeptide 9 | 125 | 156 | 163 | 2408 |
| Q6FI13 | Histone H2A type 2-A |  |  | 88 | 417 |
| Q6IQ15 | Elongation factor 1-alpha | 68 |  |  |  |
| Q6NSG7 | HMG-I(Y) |  |  |  | 83 |
| Q6NSI4 | Uncharacterized protein CXorf57 | 41 | 40 |  |  |
| Q6NZI2 | Polymerase I and transcript release factor | 82 |  |  | 166 |
| Q6P1N4 | IQGAP1 protein | 65 |  |  |  |

|  |  | **Mascot Score** | | | |
| --- | --- | --- | --- | --- | --- |
| **Accession** | **Protein Name** | **mIgG** | **HLA class I** | **Thrombin** | **bFGF** |
| Q6S8J3 | POTE-2 alpha-actin | 577 | 1339 |  | 3730 |
| Q6ZSP3 | Potential phospholipid-transporting ATPase IB |  |  | 76 |  |
| Q70EL1 | Inactive ubiquitin-specific peptidase 54 |  | 47 |  |  |
| Q71DI3 | Histone H3.2 |  | 46 |  |  |
| Q71UI9 | Histone H2A.V | 834 | 88 | 483 | 188 |
| Q86WU2 | Probable D-lactate dehydrogenase | 42 |  |  |  |
| Q86YR6 | POTE ankyrin domain family member D |  |  | 75 | 50 |
| Q8IWC5 | RGS11 protein |  | 40 |  |  |
| Q8N122 | Regulatory-associated protein of mTOR |  | 58 |  |  |
| Q8N1F8 | LKB1-interacting protein 1 |  | 40 |  |  |
| Q8N532 | TUBA1C protein | 54 | 204 |  |  |
| Q8N608 | Inactive dipeptidyl peptidase 10 | 42 |  |  |  |
| Q8N9K0 | cDNA FLJ37012 | 44 |  |  |  |
| Q8NC51 | PAI1 RNA-binding protein 1 |  |  |  | 54 |
| Q8NF91 | Nesprin-1 | 51 |  |  |  |
| Q8NFG3 | BX1 | 75 |  |  |  |
| Q8NFZ3 | Neuroligin-4, Y-linked |  | 41 |  |  |
| Q8TCG2 | Phosphatidylinositol 4-kinase type 2-beta | 78 |  |  |  |
| Q8WUM4 | Programmed cell death 6-interacting protein |  |  |  | 43 |
| Q8WZ42 | Titin | 60 |  |  |  |
| Q8WZ74 | Cortactin-binding protein 2 |  | 54 |  |  |
| Q92646 | Histone H2A | 122 |  |  |  |
| Q92945 | Far upstream element-binding protein 2 |  | 41 |  |  |
| Q96AY3 | Peptidyl-prolyl cis-trans isomerase FKBP10 | 57 |  |  |  |
| Q96B36 | Proline-rich AKT1 substrate 1 | 75 |  |  |  |
| Q96KK5 | Histone H2A type 1-H | 65 | 88 |  |  |
| Q96N16 | JAKMIP1 | 58 |  |  |  |
| Q96N66 | Lysophospholipid acyltransferase 7 |  | 52 |  |  |
| Q96NX9 | Dachshund homolog 2 | 47 |  |  |  |
| Q96PK6 | RNA-binding protein 14 | 60 |  |  |  |
| Q96QV6 | Histone H2A type 1-A | 1708 |  |  |  |
| Q99460 | 26S proteasome regulatory subunit S1 |  | 37 |  |  |
| Q99569 | Plakophilin-4 | 44 |  |  |  |
| Q99880 | Histone H2B type 1-L |  | 45 |  |  |
| Q9BQE3 | Tubulin alpha-1C chain |  |  | 578 | 52 |
| Q9BUF5 | Tubulin beta-6 chain |  | 78 | 578 | 259 |
| Q9BUF9 | Myosin, light chain 9, regulatory | 145 |  |  |  |
| Q9BV28 | TUBB3 protein |  |  | 351 | 307 |
| Q9BYZ2 | L-lactate dehydrogenase A-like 6B |  |  | 103 | 136 |
| Q9H098 | Protein FAM107B | 40 |  |  |  |
| Q9H4B7 | Tubulin beta-1 chain | 65 | 70 |  |  |
| Q9NY65 | Tubulin alpha-8 chain | 98 | 156 | 371 | 471 |
| Q9NYL9 | Tropomodulin-3 | 51 |  |  |  |
| Q9UBM7 | 7-dehydrocholesterol reductase |  | 59 |  |  |
| Q9UBM8 | MGAT4C | 56 |  |  |  |
| Q9UHP6 | Rhabdoid tumor deletion region protein 1 | 50 | 40 |  |  |
| Q9UK32 | Ribosomal protein S6 kinase alpha-6 | 76 |  |  |  |
| Q9UKX3 | Myosin-13 | 75 | 42 |  |  |

|  |  | **Mascot Score** | | | |
| --- | --- | --- | --- | --- | --- |
| **Accession** | **Protein Name** | **mIgG** | **HLA class I** | **Thrombin** | **bFGF** |
| Q9UQF2 | JNK-interacting protein 1 | 43 |  |  |  |
| Q9UQM7 | CaM kinase II subunit alpha | 56 |  |  |  |
| Q9Y2X3 | Nucleolar protein 58 |  | 117 |  |  |
| Q9Y4G2 | PH domain-containing family M member 1 | 54 | 42 |  |  |
| Q9Y4X4 | Krueppel-like factor 12 |  | 41 |  |  |
| Q9Y6X3 | MAU-2 | 42 |  |  |  |
